# Ubiquitin ligases and beyond

**DOI:** 10.1186/1741-7007-10-22

**Published:** 2012-03-15

**Authors:** Ivan Dikic, Miranda Robertson

**Affiliations:** 1 Institute of Biochemistry II, Medical Faculty of the Goethe University, University Hospital Building 75, Theodor-Stern-Kai 7, 60528 Frankfurt am Main, Germany; 2 BMC Biology, BioMed Central, 236 Gray's Inn Road, London WC1X 8HL, UK

## 

In a review published in 2004 [1] and that still repays reading today, Cecile Pickart traced the evolution of research on ubiquitination from its origins in the proteasomal degradation of proteins through the revelation that it has a central role in cell cycle regulation and the recognition of regulatory roles for ubiquitin in intracellular membrane transport, cell signalling, transcription, translation, and DNA repair.

Pickart's article marked the expansion of ubiquitination from what most regarded as a niche preoccupation, with implications only for housekeeping protein turnover and the destruction of damaged ribosomal products, to seize the attention and excite the imagination of researchers in every area of cell biology. Comparisons to phosphorylation are rife - specific ubiquitin ligases promote ubiquitination and deubiquitinating enzymes terminate its effects of ubiquitination just as ubiquitination just as kinases and phosphatases induce and terminate the effects of phosphorylation - though ubiquitination, unlike phosphorylation, can operate irreversibly, by delivering its targets to the proteasome: hence its vital role in the progression of the cell cycle.

It was already clear in 2004 that the number of ubiquitinating and deubiquitinating enzymes was very large, and that ubiquitin tags can be attached to proteins either as monomers or as poly-ubiquitin chains. But it had only recently been discovered that there are at least seven different kinds of poly-ubiquitin chains, and how the diversity of poly-ubiquitin signals is generated and interpreted in cells was in large part territory still to be explored.

In a series of articles the first three of which are published this month, we review what is now known about some of the central issues in research on ubiquitination, revisiting the questions of how ubiquitin signals are conjugated to and removed from specific targets, and how they are recognized and contribute to the regulation of central processes in cells.

## Where are we now?

**Figure 1:**
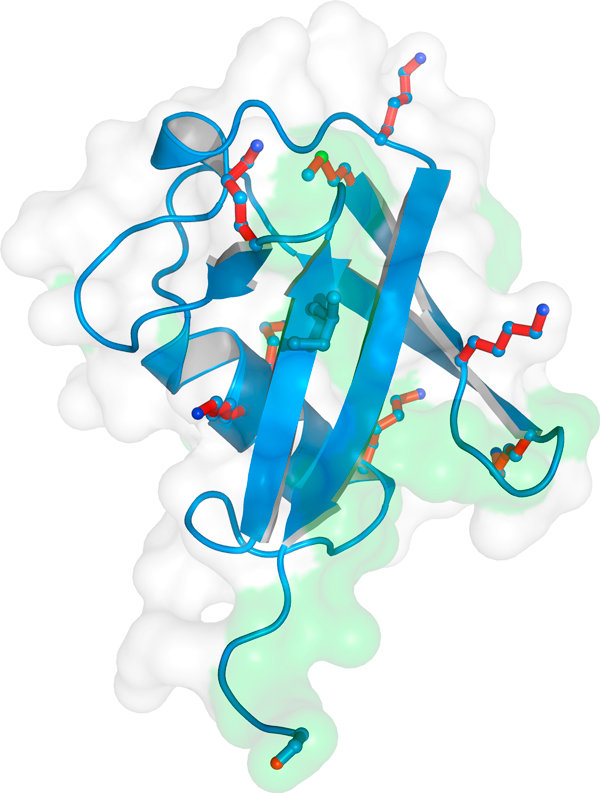
**The structure of ubiquitin**. Ubiquitin is a small, compact protein characterized by a β-grasp fold. The seven lysines that can be linked to the terminal glycine of another ubiquitin molecule to form poly-ubiquitin chains are colored red. The green shading indicates the hydrophobic patch through which ubiquitin interacts with specific ubiquitin-binding proteins. Image created by Masato Akatsu, Frankfurt University.

**Figure 2:**
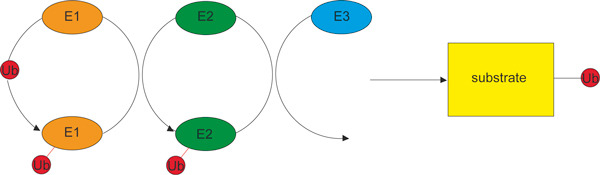
**Three enzymes act in sequence to ubiquitinate targets**. The E1 enzyme is the activating enzyme, to which ubiquitin is attached in an ATP-dependent reaction by a thioester bond (shown in red). The E2 enzyme is the conjugating enzyme, to which the ubiquitin is transferred from the E1. The E3 is the ubiquitin ligase, which directly or indirectly catalyzes the transfer of the ubiquitin to the target protein (the substrate), with the formation of an isopeptide bond (shown in black).

Ubiquitin is a protein of 76 amino acids whose structure is shown in Figure 1. It is attached to a lysine in its target proteins either as a monomer or as a poly-ubiquitin chain each monomer of which is linked through its carboxy-terminal glycine to (usually) a lysine in the preceding ubiquitin in the chain. Three enzymes, known generically as E1, E2 and E3, act in series to catalyze ubiquitination (Figure 2). The E1 is the ubiquitin-activating enzyme, to which ubiquitin becomes attached in an ATP-dependent reaction through a reactive thioester bond. E2 is the ubiquitin-conjugating enzyme, to which the ubiquitin is transferred from the E1; and E3 is the ubiquitin ligase, which binds the target protein and directly or indirectly catalyzes its ligation to the ubiquitin. The E3 therefore determines the substrate specificity of ubiquitination, and the diversity of the cellular functions of ubiquitination is reflected in the existence of some hundreds of different mammalian E3s, compared with a few dozen E2s and two E1s.

The E2 conjugating enzymes have special significance in determining the type of ubiquitin chain assembled. There are seven lysines in ubiquitin, and poly-ubiquitin chains can be assembled through linkage to any of the seven: the distinct chains are known as K6, K11, K27, K29, K33, K48 and K63 chains, depending upon the lysine through which the monomers are linked. In most cases (the so-called RING E3 ligases - see below - with are by far the most numerous) it is the E2 that decides which type of chain is made. This generalization, and the generalization that ubiquitination involves linkage through a lysine fell victim in 2006 to the discovery that ubiquitin chains can be formed through linkage between the carboxy-terminal glycine of one ubiquitin and the amino-terminal methionine of another, to form so-called linear ubiquitin chains; and in this case it is the E3 (a complex known as LUBAC) that determines the linkage (see [2]). What we now know of the mechanism of linear ubiquitin chain assembly and the function of linear ubiquitin chains in immune signaling is discussed by Henning Walczak, Kazuhiro Iwai and Ivan Dikic [2] in one of the three inaugural reviews published this month.

**Figure 3:**
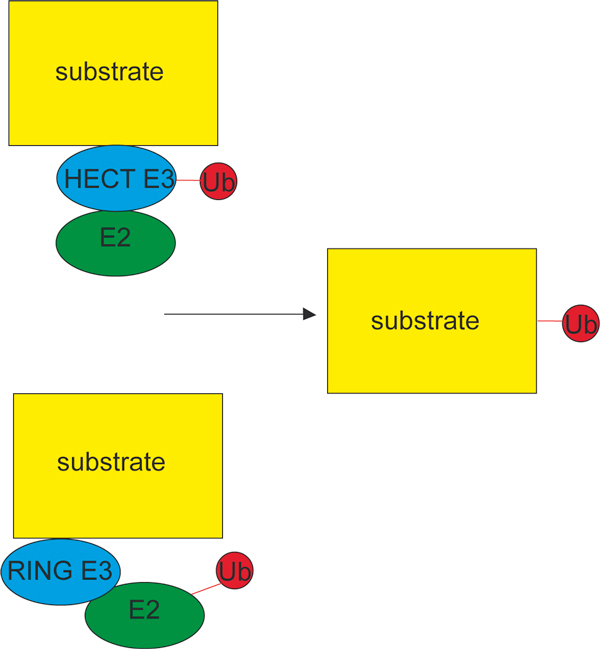
**HECT and RING ligases act by different mechanisms**. HECT E3 ligases (top) directly catalyze the attachment of ubiquitin to the substrate, whereas in the case of RING ligases (bottom) the ubiquitin is transferred from the E2 which, with the substrate, is bound to the E3. Exactly how the catalytic action of E2 is facilitated by the RING E3 is not known. Reactive thioester bonds are shown in red; the isopeptide bond with the target (substrate) protein is shown in black.

A third generalization has succumbed to research described in the second article, from Dawn Wenzel and Rachel Klevit [3]. E3 ubiquitin ligases have until recently been classified as belonging to one of two structurally and functionally distinct families: the HECT ligases, and the RING/Ubox ligases. The mechanisms of these ligases are lucidly outlined by Wenzel and Klevit and illustrated schematically in their Figure 1. Briefly, whereas in the case of the HECT ligases, the ubiquitin is transferred from the E2 to the E3, which then directly catalyzes its attachment to the substrate, in the RING ligases, the ubiquitin is transferred from the E2 to the substrate bound by the E3 (Figure 3). The RBR ligases, which are the topic of the article by Wenzel and Klevit, contain a RING domain that is structurally similar to that of other RING-type E3s, and had been regarded as a subclass of RING ligases; but it transpires that the RBR ligases behave more like the HECT family of E3s, and directly transfer the ubiquitin to the target protein. The active enzyme in the LUBAC complex belongs to the RBR subclass of E3s, helping to explain its eccentric behaviour. These fresh insights however raise again outstanding issues of how the different domains of these ligases contribute to their catalytic action and the type of chain assembled by them, and show how astonishingly little is still known about some of the fundamental mechanisms of ubiquitination.

## Beyond ligases

The world beyond the assembly of ubiquitin chains and the attachment of ubiquitin to cellular targets now encompasses all of cell biology. Ubiquitin chains of different linkages have distinct structural properties the principles of whose recognition by ubiquitin-binding proteins have yet to be fully explored. In particular, it is not known how the ubiquitin-binding modules that recognize mono-ubiquitins and the different poly-ubiquitin chains achieve the specificity for different ubiquitin species, and how this is translated into physiological responses. Recent studies have provided initial data about the mechanisms of deconjugation and functions of deubiquitinating enzymes, but many questions on the specificity of deubiquitinating enzymes and their roles in cell-specific functions remain open.

In the third review published this month, Simona Polo reaches into the territory of cell biology and explores the contribution of ubiquitination to the regulation of cell signaling by endocytosis [4], drawing parallels with the regulation at many levels of signaling pathways by phosphorylation. The critical issue here is how ligand-induced signaling regulates the activity of the E3 ligases that ubiquitinate receptors to initiate endocytic sorting for subsequent receptor degradation in the lysosome. Another important contribution of ubiquitination to the regulation of endocytosis is the ubiquitination of endocytic adaptor molecules through a process called coupled mono-ubiquitination that can be either E3-dependent and E3-independent.

Later articles in the series will confront some of the many open issues in research on ubiquitination, and extend the exploration of its contributions to fundamental cell biological processes.
